# Increase of brain Aβ peptides and secretase activity during normal aging in rodent and human

**DOI:** 10.1007/s11357-025-01926-w

**Published:** 2025-10-14

**Authors:** Jose A. Godoy-Lugo, Max A. Thorwald, Elizabeth Head, Ashley L. Gomm, Can Zhang, Rudolph E. Tanzi, Caleb E. Finch

**Affiliations:** 1 Leonard Davis School of Gerontology, University of Southern California, 3715 McClintock Ave, Los Angeles, CA 90089 USA; 2 Department of Pathology and Laboratory Medicine, University of California at Irvine, Irvine, CA USA; 3 Genetics and Aging Research Unit, MassGeneral Institute for Neurodegenerative Disease, Charlestown, MA USA; 4 Department of Neurology, Massachusetts General Hospital, Harvard Medical School, Charlestown, MA USA; 5 School of Gerontology, University of Southern California, Los Angeles, CA USA

**Keywords:** Aβ peptides, Secretase activity, Aging

## Abstract

Age increases of brain amyloid plaques may be mediated by prior increase of soluble Aβ42. Here, we show that frontal cortex samples from brains of cognitively normal aging humans had progressively increased levels of soluble amyloid peptide Aβ40 throughout the lifespan. Aggregated amyloid fraction was subsequently obtained by formic acid, where Aβ42 showed increases only in humans over 90 years old when compared to those younger than 50. Similarly, aging wild-type mice without amyloid plaques had increases of both soluble Aβ40 and Aβ42, as previously shown in normal aging rats. Aging also alters secretase enzymes and processing of amyloid precursor protein (APP). Here, we isolate membrane domains known as lipid rafts, a site of APP cleavage. We found that lipid rafts isolated from mouse and human cerebral cortex showed age increases of β-secretase enzyme activity, while amyloidogenic secretase proteins levels BACE1 and PS1 decreased with age in mouse. Lipid rafts merit further study in aging and neurodegeneration.

## Introduction

Alzheimer’s disease (AD) presents characteristic amyloid plaques and neurofibrillary tangles that arise after age 60 from fibrillar aggregations of the amyloid β-peptide Aβ42 and phosphorylated tau, respectively. A major unknown is how earlier aging alters the levels of Aβ42, the peptide that may drive amyloid aggregation. We previously showed that cerebral cortex samples of cognitively normal elderly individuals have elevated buffer soluble Aβ40, but not Aβ42 [1]. Conversely, plasma Aβ peptides are shown to increase during ages 20 to 50, followed by gradual decrease through ages 60 to 90 [2]. In contrast to blood levels, formic acid (FA)–extracted β40 and Aβ42 increase exponentially with age in frontal cerebral cortex into later ages; no data were presented for soluble Aβ [3].

Subsequent studies of wild-type rodents have also shown age increases of Aβ peptides. In Brown-Norway/Fischer rat (BN rat), buffer soluble Aβ40 and Aβ42 in cerebral cortex progressively increased across the lifespan, with an early doubling by 12 months of age, and continued Aβ42 increase at 30 months of age [4, 5]. The increase observed in BN rats, however, may partly be associated to the perivascular amyloid which is apparent at 12 months of age. Hence, our present study used the wild-type C57BL/6 mouse without detected vascular amyloid during aging [6].

**Fig. 1:**
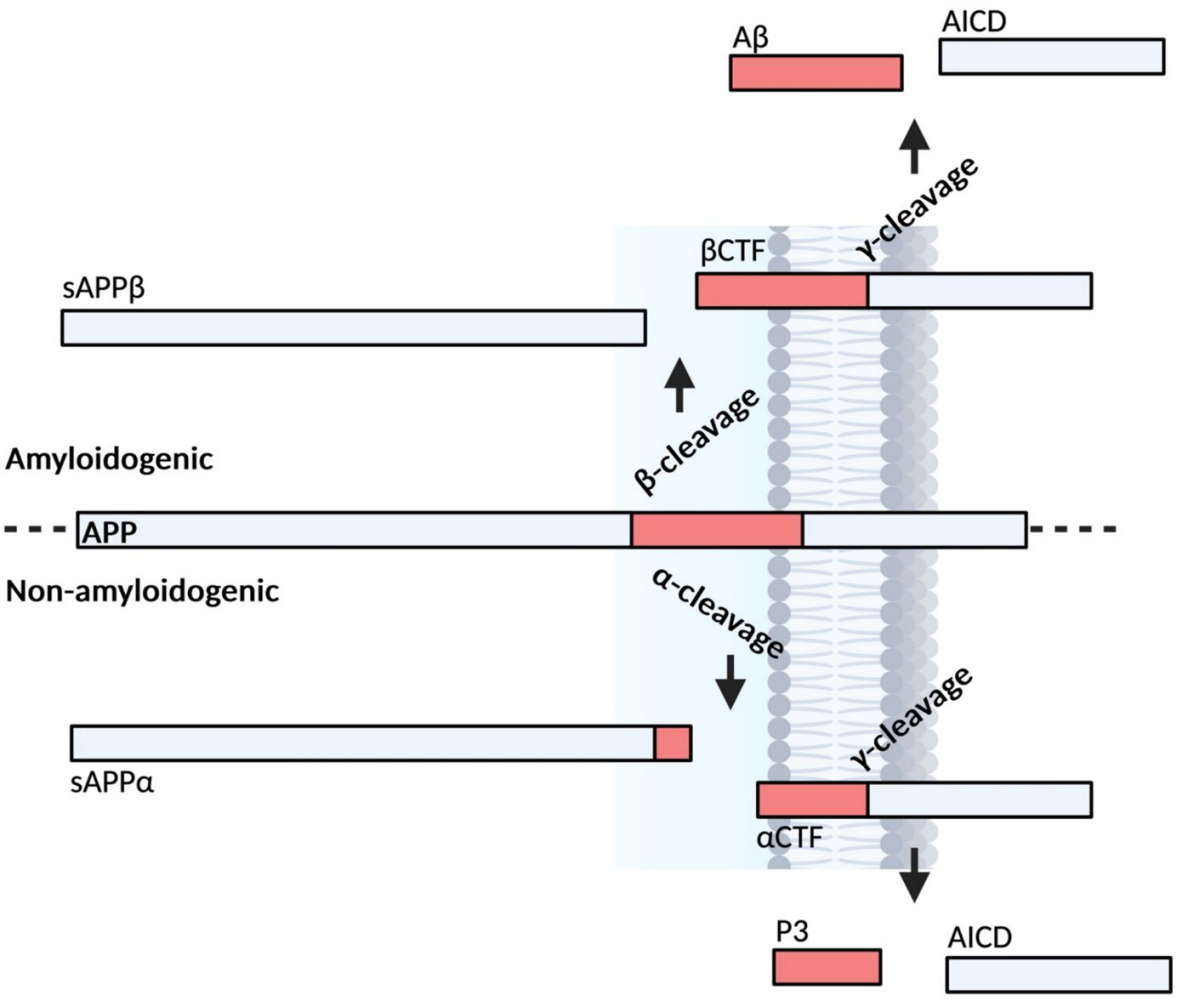
Amyloid precursor protein processing. Amyloidogenic processing follows cleavage by β-secretase and y-cleavage to produce Aβ peptides and AICD (amyloid precursor protein intracellular domain). Non-amyloidogenic α- and y-cleavage yields P38 fragments and AICD

We also introduce the use of lipid rafts to aging studies; we evaluate amyloid precursor protein (APP), the expression levels of its major isoforms, and membranous activity for the secretase enzyme that mediate amyloidogenic APP cleavage. Despite extensive study of APP processing [7–9], less is known about its subcellular cleavage. APP cleavage by the secretases is executed in two pathways: amyloidogenic and non-amyloidogenic (Fig. 1). Subcellular lipid rafts (LR), a main site of Aβ peptide production, can be altered by aging, AD, and air pollution [1, 10, 11]. LR composition changes during normal aging, with decreased total phospholipids, glycosphingolipids, and cholesterol, which decline from early adulthood into older age [12, 13]. It is suggested that composition of these LR may impact Aβ production and neurodegeneration [11]. This study measured Aβ peptides in human and mouse brain samples during normal aging, as well as APP and the secretase enzymes.

## Methods

### Human samples

Human frontal cortex (Brodmann areas 8, 9, 10) specimens from 45 cognitively normal individuals (17 females and 27 males) averaging 69 years of age were obtained from ADRC (Alzheimer’s Disease Research Center) at USC, University of California, Irvine (and the Institute for Memory Impairments and Neurological Disorders), and University of Washington. These specimens were Apolipoprotein E (ApoE) identified as 31% E4 allele carriers. Samples had an average 8 h postmortem delay, ranging from 0 to 19 h. Available cerebral amyloid angiopathy (CAA, 15% samples documented, 1% reported as positive) showed amyloid detection restricted to vessel rim in brain parenchyma; CAA positivity did not correlate with higher Aβ levels in our samples. Data for human Aβ peptide analyses (*n* = 46) include part of the previously characterized cohort (*n* = 20) [1]. All human subjects provided informed consent, all methods involving human specimens were carried following guidelines, regulations of approved IRB protocol #UP-20–00014-EXEMPT.

### Animals

All procedures were approved by the University of Southern California Institutional Animal Care and Use Committee (USC IACUC, protocol 20,417) supervised by the USC Department of Animal Resources, ensuring the ethical, humane, and appropriate care of mice. Male and female wild-type C57BL/6 J mice aged 1, 2, 4, 6 (8 mice/sex/age), 12, and 20 months (3 mice/sex/age) were obtained from Jackson Laboratories (JAX# 000664). Mice were acclimated for 2 weeks and fed ad libitum Purina Lab Chow (LabDiet, Hayward, CA) and sterile water; housed in groups of 5 at 22 °C/30% humidity and light cycles of 12 h with standard nesting material; and allowed free movement. For brain collection, mice were anesthetized with inhaled isoflurane, exsanguinated, and then perfused transcardially with PBS before decapitation. Brains were micro-dissected into corresponding regions and stored at − 80 °C.

### Amyloid β peptide extraction

Brain tissues were homogenized by motorized pestle in RIPA buffer (45 mg tissue in 120 µL) containing 1% non-ident P-40, and inhibitors of proteases and phosphatases. RIPA did not contain SDS, as it can alter the conformation Aβ forms originally extracted [14]. Homogenates were centrifuged 10,000 × *g* for 1 h at 4 °C, yielding supernatant (S1) with soluble amyloid peptides. The pellet was resuspended in 70% formic acid [15] and sonicated (50% power/10 s). After nutating for 2 h at 4 °C, the resulting suspension (S2) was sonicated again (10 s) for complete solubilization. S2 was neutralized with 20 volumes of 1 M Tris. All protein concentration was measured using Pierce 660 nm Protein Assay Reagent (22,660, Thermo Scientific). These Aβ40 and Aβ42 assays were validated for linearity exceeding the range of samples; this analysis detects Aβ peptides in the range of 0 to 250ng[1], the samples analyzed for this work were within detection range.

### Protein blotting

Western blot used 20 μg of S1 lysate resolved on 4–15% gradient acrylamide gels (Bio-Rad, Hercules, CA). Gels were transferred for 1 h at 100 V using a Criterion Blotter in an ice bath onto 0.45-μm PVDF membranes. Dot blots used 50 µg of S1 or S2 lysates were loaded into a dot blot apparatus (Bio-Rad, Hercules, CA). Loaded lysate was filtered through 0.45-μm PVDF for 2 and a half hours by gravity filtration. Membranes were stained for total protein with Revert 700. Intercept blocking buffer (Li-Cor) for 1 h before primary incubation. Antibodies for Western blot included BACE1 (D10E5, Cell Signaling Technologies), APP (ab32136, abcam), PSEN1 (16,163–1-AP) and ADAM10 (25,900–1-AP, Proteintech), sodium potassium pump (Na/K-ATPase; 3010S, Cell Signaling Technologies), and transferrin receptor (TfR; 66,180–1-Ig, Proteintech). Western blot data was presented as signal from the band normalized by the amount of protein per well, when samples were loaded in two different blots (1-month-old control mice); the average of the technical replicate was shown as a single point. Lipid raft proteins were plotted and adjusted to show presence-absence based on ratio of one defined by the average of the corresponding group. For dot blot, amyloid peptides were detected using Aβ38, Aβ40, and Aβ42 (808,601, 805,401, 805,501, Biolegend) antibodies. Membranes were visualized in the Li-Cor Odyssey Infrared Imaging System 9120 (Li-Cor) with Odyssey software, using fluorescent-conjugated secondary antibodies (Li-Cor) for analysis by ImageJ and correction with total protein stain. Representative percent change were calculated against the average of the oldest age group as 100% or calculated from two data sets measuring a group in common given increased signal of mouse amyloid peptide levels; human percent change was adjusted to a subset of older samples shared between data sets (see Human samples section).

### Quantitative PCR

Total RNA was extracted from mouse cerebral cortex using TRIzol reagents (Invitrogen), following the manufacturer’s instructions. Total RNA was treated with DNase I (Roche) to remove genomic DNA. Total RNA (2000 ng) was then used for cDNA synthesis using the High-Capacity cDNA Reverse Transcription kit (Applied Biosystems) and Oligo-dT following the manufacturer’s instructions. Quantitative reactions were run using 10 ng (equivalent total RNA) with SYBR green (Biopioneer) for APP 695 (FW-GTGGTCCGAGTTCCCACG, RV-AATGGGCATGCTCGTTCTCG), 751 (FW-TCTTTTACGGCGGATGTGGC, RV-CTGTCGTGGGAAACACGCTG), 770 (FW-CCATTCTTTTACGGCGGATGT, RV-CTTTGGGTTGACACGCTG), and HPRT1 (FW-ACATTGTGGCCCTCTGTGTG, RV-TTATGTCCCCCGTTGACTGAT) for normalization; standard curves ranged 5E − 3 to 5E − 7 ng μL^−1^.

### Lipid raft extraction

Secretase enzyme activity was measured using lipid rafts. LR was isolated from total membranes extracted from cerebral cortex, as previously shown and validated [1]. Briefly, using column-base extraction from Minute Total Lipid Raft Isolation (LR-039; Invent Biotechnologies, Plymouth, MN), 40–60 mg of brain tissue was pestle homogenized in buffer A. Columns were spun 16,000 × g for 1 min, the flow through vortexed, and spun 1000 × g for 10 min, pelleting the nuclear fraction. The supernatant was spun 16,000 × g for 30 min to recover the cytosolic fraction and pellet total membranes. Buffer B was added to the membrane pellet, vortexed and incubated in ice for 10 min. Vortexing and ice incubation were repeated two more times. The tube was spun 16,000 × g for 10 min, yielding a non-lipid raft membrane pellet. Supernatant was mixed with provided buffer C and incubated in ice for 2 min, then spun 10,000 × g for 10 min; the resulting floating membrane was then isolated. Membrane fraction and lipid raft were solubilized in PBS 1% Triton X-100 (Sigma-Aldrich).

### Secretase activity

Assay was done in 50 μg protein from freshly extracted lipid rafts from mouse and human. Förster resonance energy transfer (FRET) substrate of β-cleavage site of APP protein (20uM, 565,758, Sigma-Aldrich)[16] and α-cleavage (20uM, 565,767, Sigma-Aldrich)[17] were used. Reactions were run at pH4 (β-secretase) and pH 7 (α-secretase), incubated at 37 °C and carried out for 60 cycles, with interval reads of 60 s, as adapted from other methods [18, 19]. Inter-species comparisons were not made as murine and human β-cleavage of BACE1 differ in Aβ outputs [20]. Hence, to express β-cleavage results, percent change was calculated based as 100% to the average of each control group (4-month-old mice and to the set of younger samples in humans below age 60).

### Statistics

Groups were compared by one-way ANOVAs using Tukey’s HSD post hoc test for multiple comparisons. Significant differences for non-parametric data were calculated by Kruskal–Wallis with Dunn’s post hoc test. Outliers were calculated using ROUT (*Q* = 1%). Correlations were calculated by Pearson coefficients for age and amyloid beta and protein ratios in mouse and human.

## Results

**Fig. 2:**
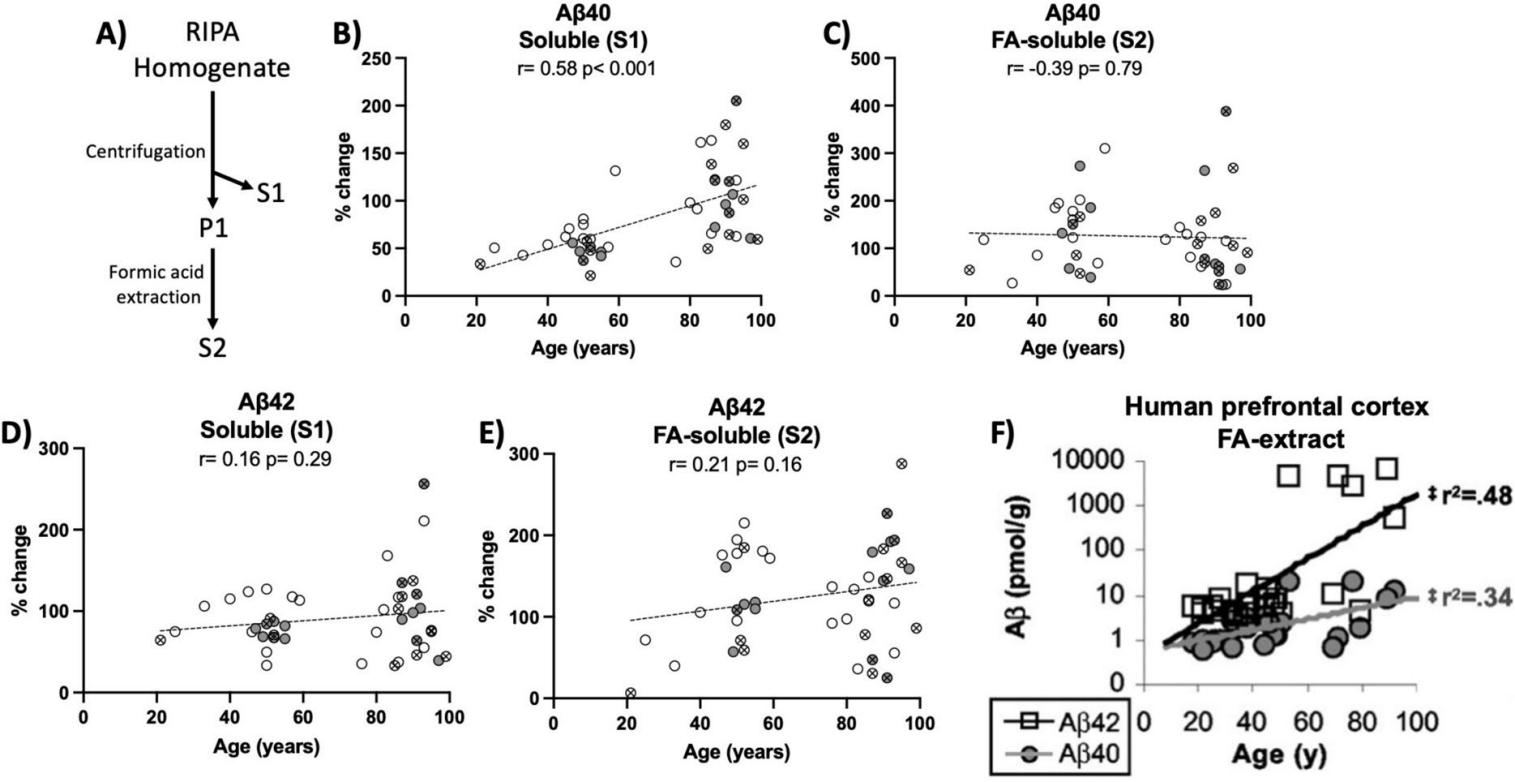
Human cerebral frontal cortex analyzed for age changes of Aβ40 and Aβ42 amyloid peptides solubilized by the sequence of RIPA followed by formic acid (FA). **A** Extraction diagram for amyloid fractions, modified from [1]. Age plots and linear regression of **B**, **D** soluble and **C**,** E** insoluble Aβ40 and Aβ42, respectively. **F** Modified panel for FA-extracted insoluble Aβ peptides from [3]. *Circles: open, male; crossed, female; shaded, ApoE4 carrier*

Soluble Aβ peptides were analyzed postmortem in cerebral cortex of cognitively normal humans and wild-type mice (WT, C57BL/6). For human samples, frozen postmortem brain tissues were fractioned for Aβ peptides (Fig. 2A) first extracted by soluble buffer (S1), followed by formic acid (FA) for the insoluble fraction (S2). Aβ peptide levels differed by age in both human and mouse.

Human samples show age increases of buffer-soluble Aβ40 (Fig. 2B), while FA-soluble Aβ40 did not change with age (Fig. 2C). Aβ42 did increase through age in either buffer- or FA-soluble extracts (Fig. 2D, E). When separated by age blocks, individuals of more than 90 years had increased FA-soluble Aβ42 compared to those below 50 years of age (Supp. Figure 1). Cerebrovascular amyloid (CAA) was considered a potential factor influencing overall Aβ peptide levels in the samples analyzed. We found CAA was not elevated in a subset of our oldest aged human samples, which may minimize potential increases in Aβ levels caused by vascular sources that may confound our results. Indeed, other factors can influence Aβ peptide levels in normal aging humans; in this study, we note that the effect of ApoE allele or sex could not be resolved because of sample size. Here we also include findings from Fukumoto et al. [3], modified and presented in Fig. 2F to illustrate the age increase of FA-soluble Aβ42 in a separate set of samples of cognitively normal humans during aging.

**Fig. 3:**
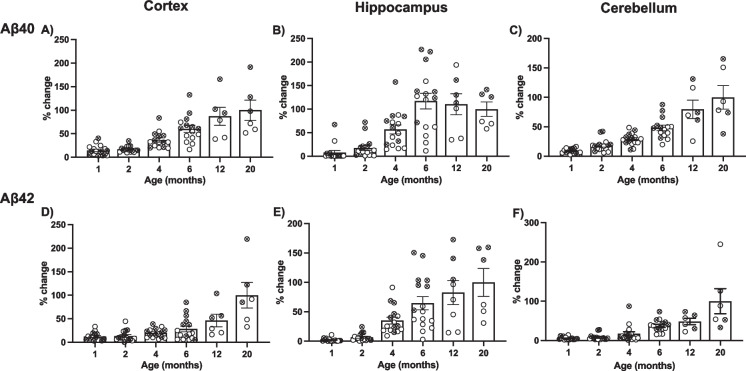
Soluble Aβ in WT mouse brain regions. Brain region levels for soluble Aβ peptides from whole cell lysate in **A**, **D** cerebral cortex, **B**, **E** hippocampus, and **C**,** F** cerebellum Aβ40 and Aβ42, respectively (Pearson correlations). *Circles: open, male; crossed, female*

Wild-type (WT) C57BL/6 J mice were evaluated for age changes in cerebral cortex, hippocampus, and cerebellum from 1 to 20 months of age. WT mice showed age increases of soluble Aβ40 and Aβ42 in cerebral cortex (Aβ40 *r* = 0.94, *p* < 0.01; Aβ42 *r* = 0.96, *p* < 0.05), hippocampus (Aβ40 *r* = 0.69, *p* = 0.1; Aβ42 *r* = 0.92, *p* < 0.01), and cerebellum (Aβ40 *r* = 0.94, *p* < 0.01; Aβ42 *r* = 0.98, *p* < 0.01) (Fig. 3). These brain regions measured shared parallel age-increases, consistent with findings of buffer-soluble Aβ from other WT rodents [4, 5, 21] and support the generality of progressive age-increase for Aβ peptides in the brain. Since Aβ can increase with age in both human and mice [5, 22], we compared the Aβ peptide levels in cerebral cortex; we found human soluble Aβ peptides were approximately 1.5-fold higher per milligram of brain tissue in early middle-aged humans compared to middle-aged mice (Supp. Figure 1); nonetheless, the differences in Aβ detection efficiency for each species may influence these results.

**Fig. 4:**
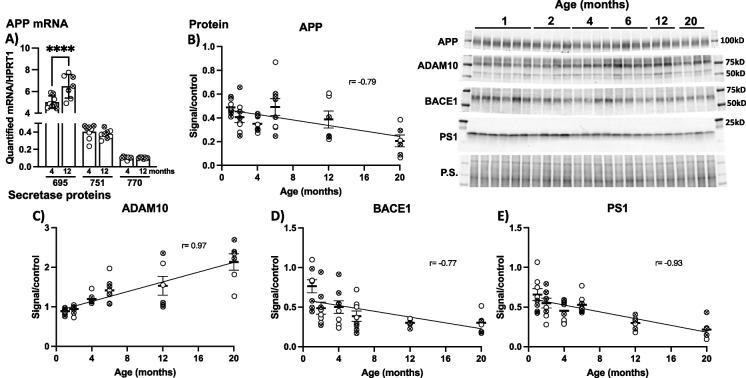
Amyloid precursor protein (APP) mRNA and protein, and APP processing enzyme protein levels in WT mouse cerebral cortex, whole cell lysate. **A** mRNA of APP isoforms 695, 751, and 770 measured by qPCR (two-way ANOVA). Protein levels for **B** APP protein and secretase proteins, **C** ADAM10 (A Disintegrin and Metalloproteinase Domain 10, **D** BACE1 (β-site APP cleaving enzyme), and **E** PS1 (presenilin 1) (Pearson correlations). P.S. = Total protein stain. Loading sequence (M = male, F = female) for APP and ADAM10 blots: M, M, M, M, F, F, M, M, F, F, M, M, F, F, M, M, F, F, M, M, F, M, M, F, and for BACE1 and PS1: M, M, M, F, F, F, M, M, F, F, M, M, F, F, M, M, F, F, M, F, F, M, F, F. Circles: open, male; crossed, female. **** p < *0.001*

Because aging increased Aβ levels in mice, we analyzed their amyloid precursor protein (APP) levels and its three major isoforms in mRNA from mouse cerebral cortex. With age, APP695 mRNA increased by 30%, whereas APP751 and 770 did not change (Fig. 4A). Basal APP695 mRNA level was higher than the other measured isoforms APP751 (tenfold lower than APP695) and APP770 (40-fold lower than APP695). For protein levels, total APP trended to decrease with age (*p* = 0.056, Fig. 4B). The secretase enzymes that cleave and process APP (shown in Fig. 1 diagram) had divergent age changes with age (Fig. 4C, D, E). The non-amyloidogenic α-secretase, ADAM10, increased with age, reaching approximately 200% by 20 months of age, compared to 1 month of age (*r* = 0.97; *p* < 0.01). In contrast, the amyloidogenic β-secretase protein, BACE1, trended to decrease with age (*r* =  − 0.77; *p* = 0.07), while the γ-secretase cleavage-site protein, PSEN1, decreased with age (*r* =  − 0.93; *p* < 0.01).

**Fig. 5:**
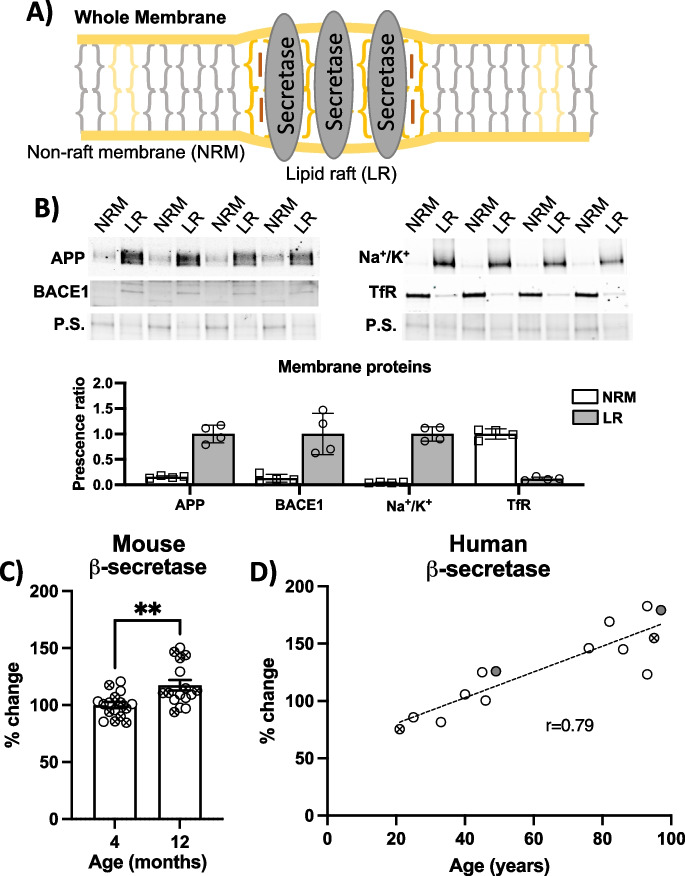
Lipid raft fractions and secretase activity in cerebral cortex of mouse and human. **A** Schematic of non-raft membrane (NRM) and lipid raft (LR), a site for amyloidogenic APP cleavage. **B** Membrane proteins and distribution to NRM or LR in mouse. **C** Mouse β-secretase enzymatic activity (*t*-test). **D** Human frontal cortex β-secretase activity in lipid raft during lifespan (Pearson correlation). P.S. = Total protein stain. Circles: open, male; crossed, female; shaded, ApoE4 carrier. ** p < *0.01*

Since Aβ levels increased in mice despite the decrease in total amyloidogenic BACE1 protein levels, we analyzed β-cleavage activity in lipid rafts from mouse cerebral cortex at young and older age. Amyloidogenic APP processing by BACE1 is described to occur at subcellular membranes [23–25]; nonetheless, the localization of this cleavage to membrane or LRs is not fully resolved [26–28]. Hence, we evaluated subcellular fractions of membrane LR and NRM for APP, the amyloidogenic enzyme BACE1 protein levels, and LR for β-cleavage activity (Fig. 5). APP and BACE1 proteins showed enrichment to LR compared to NRM (Fig. 5B). In human, APP and BACE1 were also enriched in the lipid raft, and APP protein decreased with age, while BACE1 protein levels trended to decrease (*p* = 0.06) (Supp. Figure 3). We show that despite decreasing total BACE1 levels (Fig. 4D), β-cleavage activity increased in the LR with age, specifically by 18% by early middle age compared to younger mice (4 vs 21 months of age, Fig. 5C). Accordingly, β-cleavage activity in the LR from human cerebral cortex increased by over the lifespan (Fig. 5D). We also found age did not alter LR α-secretase activity or LR yield per gram brain extracted from mouse cerebral cortex (Supp. Figure 2).

## Discussion

Because the formation of amyloid plaques depends on levels of soluble Aβ42 peptide, we evaluated the age progression of soluble Aβ peptides across the adult lifespan in cognitively normal humans with minimal Alzheimer pathology and in wild-type mice that lack fibrillar amyloid. Frontal cortex of cognitively normal humans ages 20 to 95 had minimal increase of soluble Aβ42 species, while soluble Aβ40 increased progressively. Wild-type mice, in contrast, had progressive increase of both soluble Aβ40 and Aβ42 peptides in cerebral cortex beginning earlier. In human, FA-soluble Aβ42, but not Aβ40, was increased by age. These changes to Aβ peptides were further analyzed for subcellular processing at the LR, where we found β-cleavage activity increased with age.

Amyloid peptides were extracted in two steps: Brain tissues were first homogenized in isotonic buffer with mild detergent for soluble peptides; the residue was then extracted by FA for aggregated proteins. Literature shows that insoluble amyloid peptides extracted from cerebral cortex trend to increase in aged humans[3, 22]. Samples from older aged individuals showed increased range of variation for Aβ42 levels, though a comparison between two age groups (< 50 and > 90 years of age) showed Aβ42 was increased in the FA-soluble fraction. Our correlations for age and soluble Aβ40 and FA-extracted Aβ42 levels support previous reports of age-increased FA-extracted Aβ40 and Aβ42 from human prefrontal cortex samples (included here in Fig. 2F).

With our data, the resolution of the influence of human Alzheimer’s disease risk characteristics (ApoE and sex) was not attempted, as we estimate it would require at least twice the present samples for statistical significance. The age elevation of Aβ peptides is of particular concern for ApoE4 carriers whose risk of PET-positive amyloid deposits is doubled by one allele [29]. Despite the increase in Aβ levels during normal aging, the association of amyloid burden with the pathophysiology of Alzheimer’s disease should not be disregarded[30]. Hence, clinical interventions to diminish amyloid pools could consider inhibitors of the secretases that produce soluble Aβ peptides [31, 32].

We evaluated APP levels and its processing by age. In whole homogenates of wild-type mouse cerebral cortex, the mRNA for the APP isoform 695 was the most prominent and showed 50% age increase from 4 to 12 months of age. The isoforms APP 751 and 770 lacked age differences, while their expression levels were > tenfold below APP 695 mRNA. For protein levels, total APP protein decreased progressively by twofold from 1 to 20 months old (age equivalent to human early middle-age). Secretase protein levels in these same samples had divergent directions of age change: ADAM10 increased by twofold, while BACE1 and PS1 decreased by approximately 50% by age of 20 months.

Subcellular LR fractions of human and mouse frontal cortex showed age increased activity of the β-cleavage enzyme BACE1, despite decreases in BACE1 protein levels; the same divergence was observed in other reports of β-secretase activity in human brain cortex[3]. Literature suggests that changes in LR composition may facilitate β-cleavage, thereby increasing its activity[33, 34]. Hence, studying the LR further can provide insight for regulation of β-secretase activity with aging. Such studies may be difficult, since the separation of the membrane fractions for LR and NRM depends on the isolation methods and particular reagent formulations [35–37]. The detergent-resistant LRs isolated in our work were obtained without ultracentrifugation, which may yield multiple fractions for LR and NRM. Thus, the NRM and LR resolved here were obtained in two single separate fractions and their purity was supported by selected protein markers [35]. LR was enriched for APP and mature BACE1; the NRM had minimal β-secretase activity (not shown) and no mature BACE1 protein. However, β-cleavage may not depend on BACE1 membrane localization, as shown in neuroblastoma cells with no membrane-bound BACE1, which produced equal amounts of intra- and extracellular Aβ peptides compared to untreated cells[26]. This suggests further analysis may be needed regarding APP processing for Aβ peptide production, degradation, and release. Thus, studying BACE1 dynamics within and between brain cells may guide development of drugs to target β-secretase[38, 39].

Other reports show that LR from normal aging humans have major losses of particular lipids (sphingomyelin) and fatty acids (docosahexaenoic acid) [40] that differ by brain region [13]. These membrane lipid losses in frontal cortex begin before 40 years of age and continue into later ages [12, 13]. We previously reported AD brains have lower LR yield than age-matched controls [1]. We suggest LR modifications during aging have undefined links to cognitive decline, highlighted by the impact of lipid composition in Aβ production [34, 41] through mechanisms impacting LR consistent with those observed during neurodegeneration [11]. Though age increases to amyloidogenic APP processing are reported[3, 42], we here show age-increased β-cleavage activity specific to the LR, isolated from human or rodent brains. The yield in our LR samples from cognitively normal cortex did not differ significantly with age. Future studies may further resolve mechanisms for how LR yield differs with AD, but also in other neurodegenerative conditions of aging.

Finally, we also suggest future studies consider the interaction of age-related changes to glia cells and their overlap with increased Aβ peptides. This, since APP has multiple normal cellular mechanisms involved in gene translation, mitochondrial function, nutrient metabolism, cellular signaling, proteostasis [43], and axonal growth [44]. Germline APP-knockouts mice on the C57BL/6 background are viable and deficient in memory for some tasks [45]. Moreover, neurophysiological analysis of cognitive deficits showed impaired theta-gamma coupling in hippocampus, but not prefrontal cortex at age 9 months in Cre recombinase-mediated embryo APP-KO [46]. These examples demonstrate the expanded role of APP to synapses. Thus, the study of the soluble Aβ peptide and its increase with age may be examined with brain cell-specific induced APP knockdown at different ages.

In summary, we document the progressive age changes to Aβ amyloid peptides in normal aging brains. In humans, only soluble Aβ40 increased progressively, while mice showed increased Aβ40 and Aβ42. Both human and mice showed an age increase of the APP cleaving enzyme β-secretase activity in the lipid raft, a site now described to be vulnerable to damage through environmental factors and aging [10, 11]. Future studies may identify interactions of soluble Aβ with the concurrent age decline of synapses and increased glial activity. Further findings will expand the roles of APP and add understanding of functions of APP and soluble Aβ to normal young brain physiology.

## Data Availability

Data is available upon reasonable request to corresponding author.
